# Testing the effectiveness of the Responsible, Engaged, and Loving Fathers (REAL Fathers) intervention for improving early childhood development and reducing family violence in Uganda: Study protocol for a cluster-randomized controlled trial

**DOI:** 10.1186/s13063-025-09061-9

**Published:** 2025-09-29

**Authors:** Kathryn M. Barker, Symon Wandiembe, Anslem Wandega, Joshua Jeong, Dickens Ojamuge, Rebecka Lundgren, Deogratias Yiga, Dennis Nabembezi

**Affiliations:** 1 Center on Gender Equity and Health, University of California San Diego School of Medicine, 9500 Gilman Drive #0507, La Jolla, CA 92093 USA; 2 NaNa Development Consultants Ltd, Kampala, Uganda; 3 Impact and Innovations Development Centre, Plot 175/6 Kyadondo II Road, Kagugube Zone, Makerere, P. O. Box 5150, Kampala, Uganda; 4 Rollins School of Public Health, Emory University, 1516 Clifton Road, R. Randall Rollins Building, Room 622, Atlanta, GA 30322 USA

**Keywords:** Cluster randomized controlled trial, Community-led intervention, Intimate partner violence, Violence against children, Early childhood development, Evidence-based practice

## Abstract

**Background:**

Witnessing and experiencing violence impedes children’s healthy development and learning, inhibits positive relationships, provokes low self-esteem and emotional distress, and can lead to self-harm and aggressive behavior across the life course. Evidence-based programs are needed that incorporate violence prevention strategies alongside methods to improve wellbeing and healthy development for children and their families. This trial evaluates the Responsible, Engaged, and Loving Fathers (REAL Fathers) intervention, a Ugandan-led multilevel community-based mentoring program for young fathers (ages 16–25) with children below the age of three years.

**Methods:**

To assess the REAL Fathers intervention, we use a cluster-randomized controlled trial design within 48 sub-counties randomly sampled from 24 districts in six regions of Uganda. Sub-counties were randomly allocated to treatment or control arms at baseline. Study participants are fathers ages 16–25 years and their cohabitating wives whose eldest child is below the age of 3 years (*n* = 3744 couple dyads). Primary outcomes are the following: (1) reduced intimate partner violence; (2) reduced violent discipline of children; (3) increased father-child engagement and play; (4) improved early childhood development. Secondary outcomes include the following: (1) father’s knowledge of and (2) attitudes towards positive parenting and discipline; (3) father’s use of positive parenting and discipline; (4) father’s use of emotional engagement with child; (5) couple communication and conflict resolution; (6) gender equitable household and caregiving decision-making and behaviors; (7) father engagement in child health-promoting activities; (8) improved knowledge of family planning methods; (9) reduced unmet need for family planning; (10) reduced problem alcohol use; and (11) supportive community norms for father-child engagement and play. Longitudinal survey data will be collected by trained enumerators using KoboCollect in six local languages at three time points: one month before the intervention (baseline); one month after the intervention (endline); and nine months after the intervention (follow-up). Intervention effects on primary and secondary outcomes will be assessed using difference-in-differences (DiD) mixed-effects models that account for the clustered design.

**Discussion:**

This trial will examine the impacts of a multilevel community-based intervention for young fathers and their families in Uganda on positive parenting, childhood development, and violence reduction. Overall, successful completion of this study will contribute to the evidence-based on context-informed multilevel approaches that reduce harm and promote wellbeing in families with very young children.

**Trial registration:**

ClinicalTrials.gov #NCT06100679. Registered on October 24, 2023. https://clinicaltrials.gov/study/NCT06100679.

## Administrative information

Note: the numbers in curly brackets in this protocol refer to SPIRIT checklist item numbers. The order of the items has been modified to group similar items (see http://www.equator-network.org/reporting-guidelines/spirit-2013-statement-defining-standard-protocol-items-for-clinical-trials/).

**Table:** 

Title {1}	Cluster-randomized controlled trial of the Responsible, Engaged, and Loving Fathers (REAL Fathers) intervention to assess increased father-to-child engagement and reduced family violence across Uganda
Trial registration {2a and 2b}	The trial was registered with *ClinicalTrials.gov o*n October 24, 2023. Identifier: NCT06100679. The register collects all items from the World Health Organization Trial Registration Data Set.
Protocol version {3}	Protocol Version 1.2 of December 10, 2024.
Funding {4}	The study is initiated by investigators at the Center on Gender Equity and Health at the University of California San Diego (GEH). The trial is funded by the LEGO Foundation. The investigators have no financial interests in the LEGO Foundation.
Author details {5a}	^1^Center on Gender Equity and Health, University of California San Diego School of Medicine, 9500 Gilman Drive #0507, La Jolla, CA, 92,093, USA ^2^NaNa Development Consultants Ltd, UGANDA ^3^Impact and Innovations Development Centre, Plot 175/6 Kyadondo II Road, Kagugube Zone, Makerere, P. O. Box 5150, Kampala, UGANDA ^4^Rollins School of Public Health, Emory University, 1516 Clifton Road, R. Randall Rollins Building, Room 622, Atlanta, GA 30322, USA
Name and contact information for the trial sponsor {5b}	Dr. Kathryn M. Barker: k1barker@health.ucsd.edu Center on Gender Equity and Health, University of California San Diego School of Medicine, 9500 Gilman Drive #0507, La Jolla, CA, 92,093, USA
Role of sponsor {5c}	The sponsor, LEGO Foundation, has neither participated in the design of the study, nor will it have any role in its execution; analysis and interpretation of data; or dissemination of results.

## Introduction

### Background and rationale {6a}

Violent discipline—the use of physical and psychological aggression by caregivers to punish children—and intimate partner violence (IPV) are globally the most common forms of violence experienced by children and women, respectively [1–3]. In sub-Saharan Africa, it is estimated that nearly 9 in 10 (86%) of children between the ages of 1 and 14 years have experienced violent discipline by a caregiver in the past month [4]. In Uganda, there is high prevalence among caregivers of preferences for physically harsh discipline, relative to less violent forms of child discipline [5]. Data from Uganda’s latest Demographic and Health Survey reflect these discipline preferences, indicating that 85% of children aged 1 and 14 years experienced violent discipline in the past month [6]. IPV is also common in Uganda, with nearly three in five (58%) of Ugandan women and over two in five (44.5%) of Ugandan men report experiencing some form of IPV victimization by a spouse or partner in their lifetime [6]. IPV and violent child discipline commonly co-occur in households [7, 8], with both of these forms of violence associated with immediate and long-term harms to children’s development and wellbeing [9–11]. Meta-analyses suggest a causal relationship between violence against children (VAC), witnessing parental IPV, and increased risk of experiencing and perpetrating IPV in adulthood [12–15], suggesting intergenerational linkages in violence perpetration.

We apply social network, social norms, and social learning theory to understand the ways in which VAC and IPV behaviors intersect [16–18]. These theories jointly posit that violence is learned behavior, modeled by important individuals within peer groups, families, and communities [19, 20]. For example, empirical evidence has shown that violence is “socially contagious” and clusters within households [21, 22]. Previous work in Uganda shows that both boys and girls who witnessed and experienced violence in their homes had between 3.23 (95% CI 1.99 to 5.24) and 8.12 (95% CI 5.15 to 12.80) times the odds of using physical or sexual violence themselves [23]. Exposure to physical punishment during childhood is correlated with perpetration of similar practices toward one’s own children later in life [14, 24]. Studies confirm multiple ways that VAC and IPV intersect, including the following: shared risk factors such as gender inequality and marital conflict; social and gender norms that condone violence and promote masculinities based on violence and control; clustering of VAC and IPV within the same households; intergenerational transmission; and shared consequences of VAC and IPV on social, emotional, and physical wellbeing [8, 22]. These consequences include harms to the following: physical and mental health [25]; cognitive, social-emotional, and behavioral development [26]; and educational outcomes [27].

Given the many intersections between IPV and VAC, a growing literature has examined coordinated responses to prevent the two forms of violence [28, 29]. Emerging evidence indicates the importance of parenting programs to address violence against children and women [30]; however, few parenting programs explicitly seek to reduce both IPV and VAC in joint programming, and many either focus solely on female caregivers or find it challenging to recruit and retain male caregivers [31, 32]. Working with both female and male parents and caregivers capitalizes on the important role that fathers play in shaping positive child development outcomes, including social, emotional, and cognitive development [33, 34]. Engaging both caregivers also contributes to the transformation of restrictive and harmful social and gender norms that are known shared risk factors for both IPV and VAC [35]. Further, in addition to reducing and preventing IPV and VAC incidence, there is a need to strengthen protective factors against later-in-life harms and intergenerational cycles of violence [36]. Parent–child connectedness and attachment, as well as healthy and empathic communication between intimate partners, are increasingly recognized as protective factors against intergenerational IPV and VAC [37–39].

#### The REAL Fathers Intervention

This trial seeks to evaluate the Uganda-based intervention known as Responsible, Engaged and Loving Fathers (REAL Fathers), a multilevel social and behavior change intervention that trains community-based mentors to work with young fathers (ages 16–25 years) and their female partners on positive parenting and communication skills-building [40]. The intervention’s mentors hold home-based individual mentoring and community-based group mentoring sessions with young fathers and spouses, alongside project-level community engagement opportunities. The intervention is designed to address common antecedents of VAC and IPV, including harmful social and gender norms, or the unwritten rules, values, and expectations of individuals within a community that condone violence, promote gender inequality and discrimination, and promote masculinities based on violence and control [8, 41]. The intervention components are grounded in social learning theory [42] and social cognitive theory of gender development and differentiation [43]. The REAL Fathers intervention uses modeling of alternative strategies for nonviolent discipline and conflict resolution to improve fathers’ parenting and communication skills and confidence in adapting nonviolent strategies, with related shifts in gender norms to expand the socially acceptable behaviors available to men as they self-regulate to adhere to gender roles and expectations in their communities.

From its inception, REAL Fathers has drawn upon cross-sector partnerships to address community needs and to promote scale and sustainability. The intervention was developed by communities in Northern Uganda in partnership with Save the Children and Georgetown University [40]. Piloted in 2013 in post-conflict northern Uganda (Amuru District), it was proven effective in reducing violence via mentorship on positive parenting, non-violent discipline, and gender equity and respectful couple communication [40]. REAL Fathers was subsequently scaled in additional areas of the Northern Region (Nwoya, Gulu, and Nakapiripirit) in 2020, with the involvement of the original program designers, implementers, and researchers. In this same period, funding was also provided to identify stakeholders and mechanisms to adapt, scale, and implement REAL Fathers in Uganda-based early childhood development (ECD) programs and centers. In January 2022, the Government of Uganda endorsed REAL Fathers as an implementation strategy in support of its third National Development Plan (NDP III 2021–2025) and related early childhood development policies and frameworks. With this endorsement and government buy-in, the partnership submitted and was awarded a grant to scale REAL Fathers to reach 280,000 fathers and 425,000 children from 2023–2027 in six new regions across Uganda: Ankole, Buganda, Bunyoro, Busoga, Lango, and Teso. It is not feasible or desired to reach the planned number of beneficiaries (i.e., fathers and children) simultaneously (i.e., all at once). As such, there will be five rounds of REAL Fathers implementation over the course of the four-year project. As depicted in Table 1, we will embed this present trial into the current scale-up of REAL Fathers, using a subsample of fathers involved in the wider intervention.

**Table 1:**
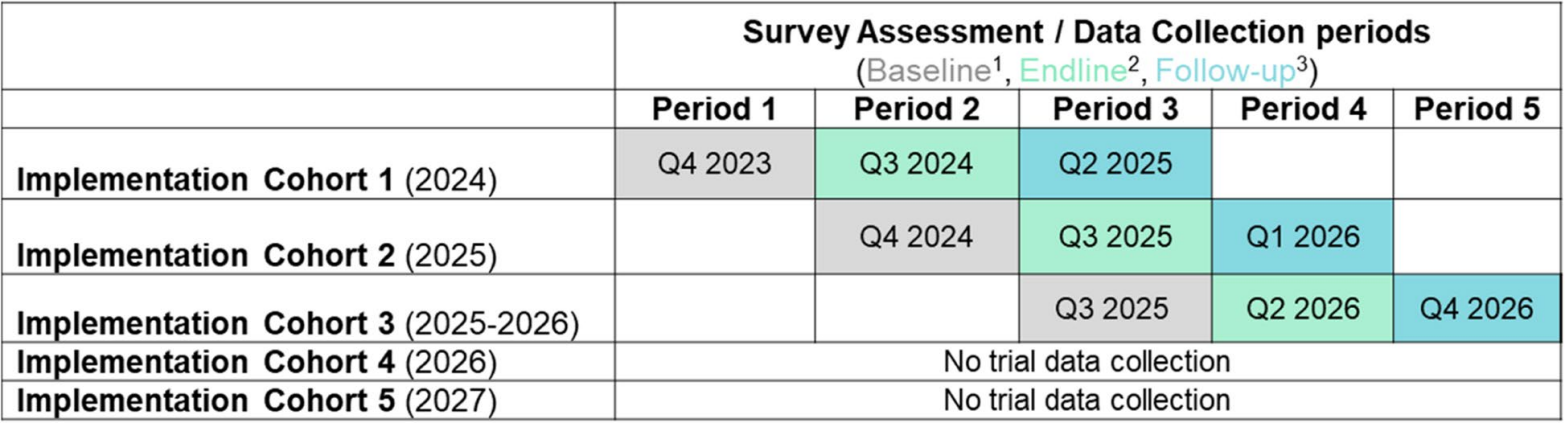
Mapping of trial assessment periods on REAL Fathers implementation cohorts

### Objectives {7}

**Fig. 1:**
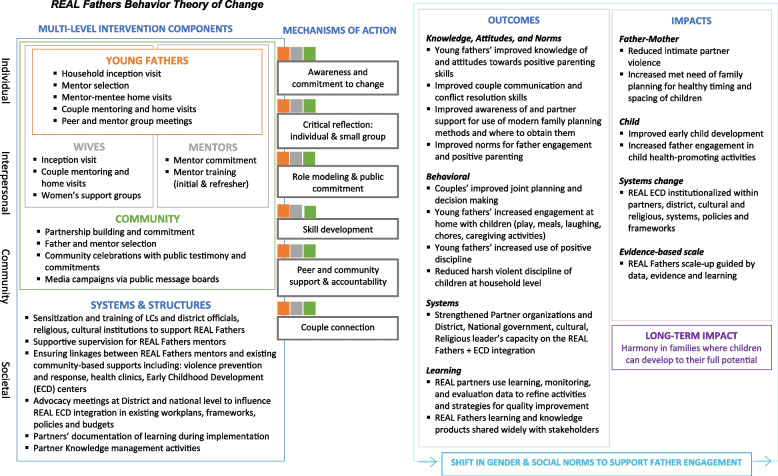
REAL Fathers behavior theory of change

The present trial aims to test the effectiveness of the REAL Fathers intervention in reducing rates of family violence and improving child wellbeing and development outcomes in six regions of Uganda that have not yet received the REAL Fathers intervention. We hypothesize that, as compared to study participants in the waitlist control group who receive standard care, participants in the intervention group will see improvements in the primary and secondary outcome measures (as listed below). Activity inputs, mechanisms of action, outputs, and outcomes are summarized in Fig. 1 (*Theory of Change—end of document*).

### Trial design {8}

The impact of the REAL Fathers intervention will be assessed using a cluster-randomized controlled trial (cRCT) wait-list control rollout design [44]. This cRCT employs a superiority framework to test the null hypothesis that there will be similar differences in the primary and secondary outcomes from baseline to endline in both the REAL Fathers intervention group and the waitlisted control group receiving standard care [45]. The survey questionnaire will use validated questions, as piloted in a previous evaluation of the intervention [40], and will be extended to include items from Caregiver Reported Early Development Instruments (CREDI) [46] and socio-demographic items adapted to the local context. The phased rollout of the study will involve three cohorts, each with 1872 father-spouse dyads in the control group and 1,872 father-spouse dyads in the intervention group, for a total of 3744 father-spouse dyads across the three cohorts and study arms. Baseline data collection will occur roughly one month before the start of a cohort, with endline data collection occurring roughly 8 months later (i.e., one month after the 7-month mentoring period of the REAL Fathers intervention ends). Follow-up data collection will occur 8 months after endline data collection. This rollout design is optimal for resource constraint and ethical considerations. The trial is designed to match the speed of implementation, with a random sample of study participants in the intervention group selected from the overall implementation cohorts. Due to personnel and resource constraints in reaching the large number of program beneficiaries, staggered implementation of the intervention—and therefore also the cRCT trial which uses a sub-sample of program beneficiaries—is necessary. Further, from an ethical standpoint, in order to offer those in the control conditions of the study an opportunity to participate in REAL Fathers, several implementation cohorts are necessary, making this wait-list control rollout design the most appropriate for the study context.

## Methods: participants, interventions, and outcomes

### Study setting {9}

**Fig. 2:**
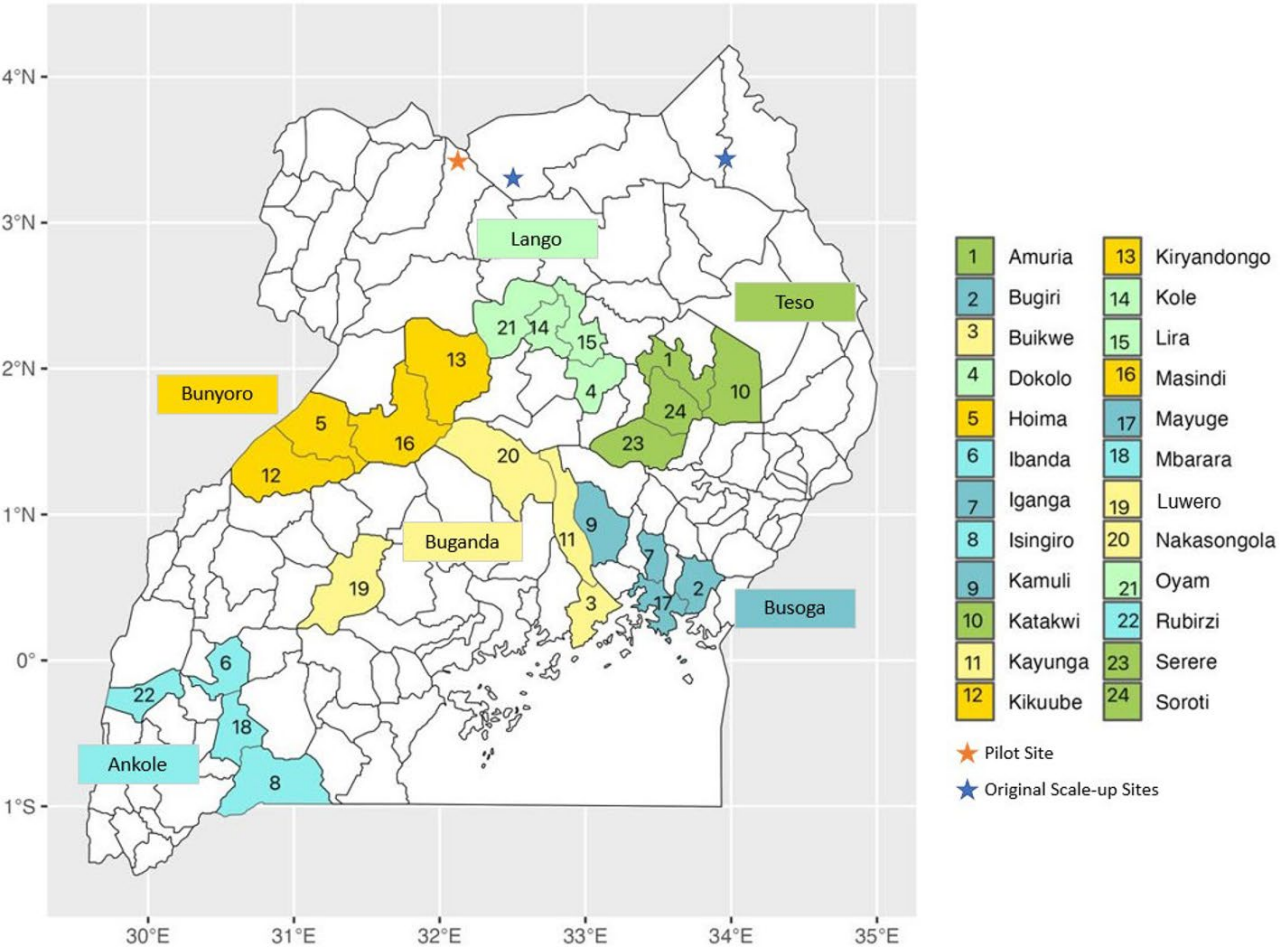
REAL Fathers implementing districts in Uganda

The study will be conducted in 24 districts across six regions of Uganda (four districts in each region—Fig. 2). The regions and districts include those not previously reached in pilot trials of the REAL Fathers intervention and are as follows:

1) Ankole Region: Ibanda, Mbarara, Isingiro, and Rubirizi districts [Implementing Partner: FAWE Uganda Chapter]

2) Buganda Region: Kayunga, Buikwe, Luweero, and Nakasongola districts [Somero Uganda]

3) Bunyoro Region: Hoima, Kikuube, Kiryandongo, and Masindi districts [Bantwana Initiative Uganda]

4) Busoga Region: Kamuli, Bugiri, Iganga, and Mayuge districts [Somero Uganda]

5) Lango Region: Dokolo, Kole, Lira, and Oyam districts [All Nations Child Development Centre]

6) Teso Region: Amuria, Katakwi, Serere, and Soroti districts [Bantwana Initiative Uganda]

Community and village leaders within these 24 districts will be contacted by implementing partner staff (i.e., staff of the not-for-profit community-based organizations leading the implementation of the REAL Fathers intervention) to share information about REAL Fathers and to gauge the community leaders’ interest in and support for the intervention.

### Eligibility criteria {10}

Trial eligibility will be based on a two-stage screening process. In stage 1, project officers of the implementing partner organizations will contact local leaders (e.g., local civil servants, clan leaders, religious leaders) to share information about REAL Fathers and to generate a list of communities expressing interest in and support for their communities to become involved in REAL Fathers. In stage 2, the research team will conduct a listing/census exercise within the interested communities to identify all young men between the ages of 16 and 25 years. As summarized in Table 2, trial-eligible young men will be between the ages of 16 and 25 years; a permanent resident of the village; able to speak English or one of the six local languages; cohabitating with or married to a female partner; and have at least one child who is not over the age of three years. Only couples where both the father and his female partner consent to participate will be eligible to participate in the study.

**Table 2:** Inclusion criteria by respondent type

		**Male respondents**	**Female respondents**
1	Between the ages of 16 and 25 years	✓	*No age restriction*
2	Permanent resident of one of the 24 districts (i.e., not a migrant or refugee)	✓	✓
3	Speaks English or one of the local languages (Runyoro, Langi, Luganda, Lusoga, Runyakitara, Ateso)	✓	✓
4	Biological parent to a child(ren)	✓	*Can be a guardian*
5	Oldest biological child under the age of 3 years	✓	✓
6	Living in the same household with the child(ren)	✓	✓
7	Living in the same household with the other parent (or guardian) of the child(ren)	✓	✓
8	Living in union with the mother of the child(ren) intimate partner	✓	*NA*
9	Spouse/partner consented to participate	✓	✓

### Who will take informed consent? {26a}

Given REAL Fathers emphasizes the role of fathers, we will first assess the eligibility of fathers in screening interviews. Eligible fathers will be invited to participate in the study and subsequently scheduled to participate in an interview. After fathers are invited and consented, their female partners will subsequently be invited to participate. All respondents will provide digital signature on the data collection tablets. A model consent form that will be programmed into KoboCollect is provided in the Appendix.

### Additional consent provisions for collection and use of participant data and biological specimens {26b}

This trial does not involve collecting biological specimens for storage.

## Interventions

### Explanation for the choice of comparators {6b}

Random allocation is made between intervention and wait-list control groups, as randomized at the sub-county level. The intervention group will receive the REAL Fathers intervention within a month after the screening process, while the wait-list control group will receive the REAL Fathers intervention after follow-up data collection (wave three) is conducted. Participants in the control group will continue to access standard care and services that are already available in their communities. These include the following: village health team visits and referrals to clinics when applicable; early childhood development centers and programming; family planning and social-economic interventions from Government, as described in further detail in the “Plans to promote participant retention and complete follow-up” section below.

### Intervention description {11a}

**Fig. 3:**
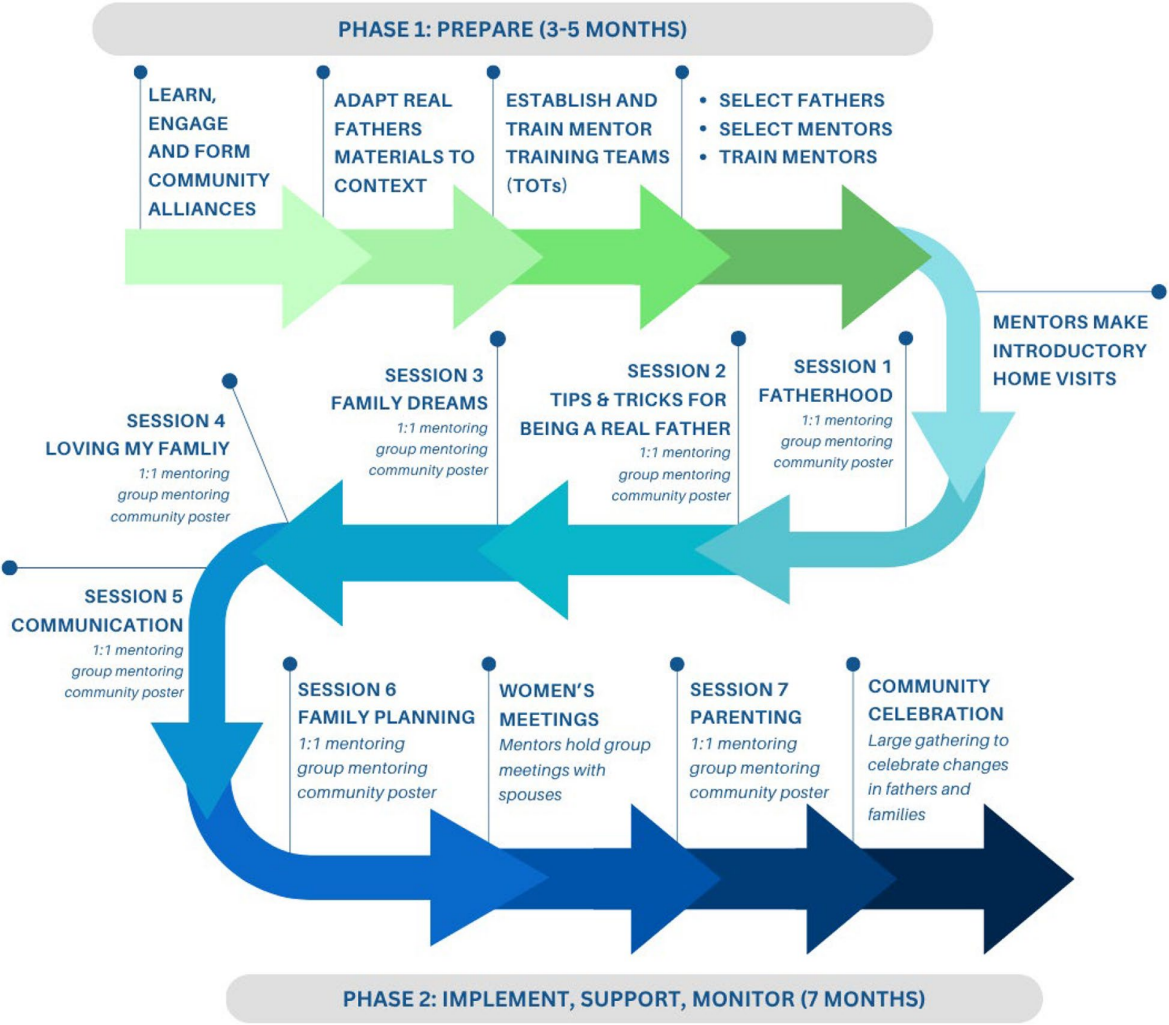
The REAL Fathers intervention at a glance

In acknowledgement of the critical nature of community support for the implementation of the REAL Fathers intervention, the depiction of the REAL Fathers intervention timeline in Fig. 3 includes a set of preparation activities that precede the mentoring components of the intervention, but that are integral to laying the groundwork for successful mentorship: community sensitization and engagement; adaptation of materials to the local context; and training of local community members. The second phase, Implement, Support, and Monitor, involves one-to-one and group mentoring of fathers, their spouses, community engagement and support, and adaptive management and complexity-aware monitoring.

Phase 1 involves community sensitization, awareness, and advocacy meetings to learn about the intervention at either the village or parish level. Meetings cover what the REAL Fathers intervention does and aims to do, the target population, objectives and community roles, and eligibility criteria for participation in the intervention. In such meetings, some young fathers who fit the criteria are naturally identified and volunteer to be recruited. Other young fathers are identified by people in the meeting who they think fulfil the criteria. These young fathers are contacted by the project officers for further screening and recruitment into the intervention. After identifying interested young fathers, the young fathers are asked to nominate older fathers in their communities who they view as upstanding citizens and respected role models. Prospective mentors are contacted, informed of the study, and invited to participate. After selection, mentors undergo a five-day training on a variety of topics, as summarized in Table 3.

**Table 3:** Content for training of mentors

Topic	Session
**Understanding REAL Fathers, Early Childhood Development and Trainer skills**	Introductions, ground rules and agenda
	Training of Trainer competencies and skills
	Introduction to REAL Fathers and Research Study
	Introduction to Early Child Development
	Components of Nurturing Care for Child Development
	Child Development Stages & Developmental Needs of a Child
**Understanding Values and Norms**	Gender Values Clarification
	Gender Roles and Household Decision-Making
	Gender Norms: Behave Like a Man, Behave Like a Woman
	What Kind of Husband and Father Am I?
**Happy Fathers, Happy Children**	Tips and Tricks to Being the Best Father in the Village
	In It to Win It: How Fatherhood Is Like Football
	Disciplining Your Child with Love
	From My Child’s Perspective
	The Invisible Wall
	Things Your Child Needs to Hear You Say
**Happy Homes: Living in Peace**	Why Do We Act This Way?
	Dealing with Stress and Managing Our Emotions
	Don’t Talk to Me That Way!
	Stronger Couples through Communication
	Good Sportsmanship: How the Rules of Marriage Are Similar to a Game of Football
**Safeguarding**	Definition and Examples of Abuse in Mentor–Mentee Relationships
	Safe Mentorship: Preventing Abuse and Referral Process
	Legal and Cultural Contexts of Safeguarding
	Mentor Code of Conduct
**Practicum: Putting It All Together**	Home Visit Protocol
	Home Visits with Young Fathers
	Home Visits with Couples
	Group Meetings Discussion Guide
	Women’s Group Session Guide

Following successful completion of the 5-day training, mentors then work directly with the young fathers who nominated them as mentors (up to 3 fathers per mentor) to provide 7 monthly individual and group mentoring sessions (one individual and group session per month), lasting roughly 1 to 1.5 h each. The mentoring sessions cover similar topics as those covered in the mentor training (see session titles in Fig. 3, with full details on session curricula provided elsewhere [47]). These individual, couple-, and group-based mentoring sessions are coupled with a community poster campaign and community celebration at the end of the seven sessions to model alternative strategies for nonviolent discipline and conflict resolution to improve fathers’ parenting and communication skills and confidence in adapting nonviolent strategies. The intervention is being implemented by four Uganda- and community-based organizations that are independent of the evaluation team. These include the following: Somero Uganda; All Nations Child Development Centre; Bantwana Initiative Uganda; and Forum for African Women Educationalists Uganda Chapter.

### Criteria for discontinuing or modifying allocated interventions {11b}

Given the nature of the intervention, we do not anticipate adverse events related to its delivery that will lead to termination of the trial. As such, there are no criteria in place for trial termination or for modifying intervention allocation. The REAL Fathers Consortium will apply responsive feedback mechanisms as outlined elsewhere [48], to allow systematic modifications to the intervention components between cohorts. These cohort effects will be accounted for in subsequent difference-in-differences analyses.

### Strategies to improve adherence to interventions {11c}

Adherence to the intervention (i.e., implementation fidelity, the degree to which the intervention is delivered as intended) will be assessed and supported through ongoing monitoring data collection by both the mentors and the implementing partner staff via a series of monitoring tools. Mentors will collect data on participation of fathers in the individual and group sessions, as well as the quality of the session. Mentors will also have access to the fathers’ phone numbers to help facilitate scheduling and follow-up.

Implementing partner staff will engage in regular check-in meetings with the mentors during the mentoring sessions to not only document what the mentors cover in the session, but to also provide supportive supervision and engage in standardized feedback on the session to improve implementation fidelity. All data will be uploaded to an online Monitoring Information System (MIS), with reports generated to respond to specific monitoring indicators. Using the MIS reports, the Monitoring and Evaluation officers at each implementing partner (IP) will review the monitoring data collected each month and provide monthly monitoring updates to the IP management.

Routine Data Utilization meetings will be held by the M&E officers at each implementing partner among all project officers to engage in review of the data and dialogue on the realities experienced in the communities that reflect what’s shown in the data. This regular exchange will encourage program officers to ensure each monthly session is being delivered as intended and to identify specific mentors or communities that may need additional support. Project officers will also meet with mentors on a monthly basis to conduct supportive supervision exercises and to provide feedback on the extent to which the mentors delivered the session material as planned. Finally, although no financial incentives are provided to the mentors, social status will be gained via increased visibility in the community and involvement in local leadership. This elevation in status of the mentors will incentivize the mentor fathers to stay continually active in their mentoring activities.

These reports will provide regular updates on the progress of each implementing partner on maintaining adherence to the protocol and schedule of the intervention. In addition, the managing organization will conduct routine supportive supervision for the implementing partners to review progress and provide feedback on areas for improvement. In the event that a mentor and/or the fathers are not engaging in the intervention material as planned, meetings will be set to follow up with the mentors and fathers to understand the challenges they may be facing.

### Relevant concomitant care permitted or prohibited during the trial {11d}

No concomitant care prohibited.

### Provisions for post-trial care {30}

Not applicable; no risk of harm anticipated; no provisions prepared. In the event of potential harm, participants will be encouraged by mentors to seek support from health and social workers trained in violence prevention and harm reduction.

### Outcomes {12}

This trial is designed to assess differences in the specified outcome measures between the intervention and waitlist control groups at endline and follow-up. All primary and secondary outcomes will be assessed at three points: (1) Baseline, one month before the intervention; (2) Endline, one month after the intervention ends (nine months after the baseline survey); and (3) Follow-up, 9 months after the intervention ends (17 months after the baseline survey).

#### Primary outcomes

The study’s primary outcomes (Table 4) are reductions in intimate violence, violence against children, increased father-child engagement (early stimulation), and improvements in early childhood development outcomes.

**Table 4:** Summary of primary outcome measures

Primary outcome	Measure description	Questionnaire
Intimate partner violence (IPV): Perpetration and Victimization	Proportion of young fathers and their wives reporting perpetrating or being a victim of physical, sexual, and/or emotional IPV in the past three months. Assessed via six measures: insulted; shouted or yelled; slapped; pushed or shoved; threw something at that could hurt; physically forced unwanted sex	Male and Female caregiver
Harsh violent discipline of children	Proportion of fathers reporting they used a form of harsh/violent discipline with their child in the past month. Items include the following: shook child; shouted, yelled at, or screamed at child; spanked, hit, or slapped child on the bottom with bare hand; hit child on the bottom or elsewhere on the body with something like a belt, stick, or other hard object; hit or slapped child on the face, head, or ear; hit or slapped child on the hand, arm, or leg; beat child up, that is, hit him/her over and over as hard as you could	Male
Fathers’ increased use of child engagement (early stimulation) activities	Proportion of fathers reporting use of early learning and play activities with child (i.e., stimulation activities). Activities include the following: reading, telling stories, singing songs, taking child outside of the home, naming/counting things together, drawing things together, playing with a toy or object	Male
Early childhood development	Scores on the short-form version of the Caregiver Reported Early Development Instruments (CREDI) [46]	Male and Female

#### Secondary outcomes

The secondary outcomes (Table 5) include hypothesized antecedents to the primary outcomes with respect to knowledge, attitudes, and behaviors: improved couple communication, increased father engagement in household duties; improved father knowledge and attitudes regarding positive discipline; increased father use of positive discipline; and increased father engagement in health-promoting activities for children. In addition, norms measures are included to assess supportive normative social environments for father engagement and positive parenting, and family planning knowledge and behaviors are included to understand potential connections between father engagement, couple communication, and use of contraceptives.

**Table 5:** Summary of secondary outcome measures

Secondary outcome	Measure description	Questionnaire
Couple communication and conflict resolution	Proportion of fathers and mothers reporting increased and improved communication with the other. Questions include the following: past month took time to listen to partner’s concerns; communicated with each other about things that frustrated you; talked about things that affect each other and the family; and asked questions and got feedback/input from each other before making final decisions; resolved conflicts/arguments amicably without physically hurting each other	Male and Female
Father engagement in household duties	Proportion of fathers who engage with household chores and caretaking activities in past three months. Items include the following: washed clothes; prepared food; bought food; cooked or fixed food for your child; dressed or changed the clothes of the child; gave your child a bath	Male
Father alcohol and drug use	Reduction in proportion of fathers’ heavy alcohol use (i.e., three or more drinks in a single day)	Male
Fathers’ use of emotional engagement towards child	Proportion of fathers reporting increased emotional engagement and affection towards children. Activities include the following: interact in a calm/gentle manner, use affirming language, recognize when they need to be comforted, use words of endearment, show approval, ask questions to engage with the child	Male
Fathers'knowledge of positive parenting and discipline skills	Proportion of fathers who can correctly distinguish between positive and harsh discipline. The list of discipline behaviors included in the survey as true/false questions are as follows: Taking a deep breath (calming yourself) when the child does something wrong before responding; Threaten to hit child; Tell your wife to take care of the child; Put child somewhere by him/herself; Redirect child's attention or behavior; Ask your wife for advice or help; Shout at child	Male
Fathers'attitudes towards positive parenting and discipline	Proportion of fathers who hold positive attitudes for the use of non-violent parenting practices. Items include the following: Stubborn children need to be hit to teach them right from wrong [reverse-coded].; A parent should never spank or hit a child.; Being violent with my child(ren) is not the only way to be an effective head of the family.; If a child is old enough to defy a parent, then he/she is old enough to be hit. [reverse-coded]	Male
Fathers'positive parenting and discipline behaviors	Proportion of fathers reporting use of positive parenting and discipline practices in the past three months. Questions include the following: whether the father said something nice about or praised the child; gave the child physical affection; went someplace or did something special with the child as a reward; showed or told the child that you love him/her. Discipline strategies include the following: taking away something the child liked; explaining why the child's behavior was wrong; redirecting (giving the child something else to do); asking the child to apologize	Male
Supportive environments for father engagement and positive parenting	Proportion of fathers agreeing or strongly agreeing that their social networks approve of engaging with children and using positive parenting skills. Items include the following: Your closest friends will tease you if you help your wife with child care. [reverse-coded]; Your father would admire you for telling stories to your child(ren).; You would be embarrassed if your mother saw your child(ren) misbehave and you did not discipline the child strongly. [reverse-coded]; Your friends think you should discipline your child(ren) with love.; Your respected community members will say you are not parenting your child well if—instead of beating your child—you try to explain to them their misbehavior. [reverse-coded]	Male
Father engagement in child health-promoting activities	Proportion of fathers and mothers reporting that the father took their child(ren) for rounds of immunization(s)	Male and Female
Knowledge of where to access family planning method(s)	Proportion of fathers and mothers who know where to obtain a method of family planning	Male and Female
Reduced unmet need for family planning	Proportion of fathers and mothers with unmet need for healthy timing and spacing of children. Assessed via those who are fecund and sexually active but are not using any method of contraception, and report not wanting any more children or wanting to delay the next child for 2 or more years	Male and Female

### Participant timeline {13}

This cRCT will roll out in a phased manner in conjunction with the larger implementation project using a subsample of individuals in three of the five implementation groups included as the intervention group in the trial. Sufficient time is planned to allow waitlist controls to transition to intervention groups in subsequent implementation rounds after they participate in the follow-up assessment. As shown in Table 6, for each trial cohort, baseline data collection will occur roughly one month before the start of a cohort, with endline data collection occurring roughly 8 months later (i.e., one month after the REAL Fathers intervention group ends). Follow-up data collection will occur 8 months after endline data collection.

**Table 6:** Trial schedule: Enrolment, intervention, and assessments (adapted from SPIRIT 2013 Figure [36])

Date	Trial activity	Intervention activity		Cohort		
		REAL Fathers	Standard care	1	2	3
Nov 2023	Cohort 1 (C1) enrolment: eligibility screen and informed consent	Community engagement and sensitization (Steps 1–3)		1		
Dec 2023	C1 baseline assessment	REAL Fathers materials adaptations (Step 4) & Training of trainers (Step 5)		1		
Dec 2023 (after baseline)	Randomization (allocation to control or intervention)	Selection and training of mentors; selection of fathers (Steps 6–9)	x	1		
Jan–Aug 2024		Individual and group mentoring; community posters (Steps 10–11)	x	1		
Oct–Nov 2024	C1 endline assessment (1 mo. after intervention ends)			1		
Dec 2024–Jan 2025		Pause and Reflect				
Jan–Feb 2025	C2 enrolment: eligibility screen and informed consent	Community engagement and sensitization (Steps 1–3)			2	
Feb–Mar 2025	C2 baseline assessment	REAL Fathers materials adaptations (Step 4) & Training of trainers (Step 5)			2	
Mar 2025 (after baseline)	Randomization (allocation to control or intervention)	Selection and training of mentors; selection of fathers (Steps 6–9)	x		2	
Apr–Nov 2025		Individual and group mentoring; community posters (Steps 10–11)	x		2	
Jul 2025	C1 follow-up assessment			1	2	
Dec 2025	C2 endline assessment (1 mo. after intervention ends)				2	
Jan 2026	C3 enrolment: eligibility screen and informed consent	Community engagement and sensitization (Steps 1–3)				3
Feb 2026	C3 baseline assessment	REAL Fathers materials adaptations (Step 4) & Training of trainers (Step 5)				3
Feb 2026 (after baseline)	Randomization (allocation to control or intervention)	Selection and training of mentors; selection of fathers (Steps 6–9)	x			3
Mar–Oct 2026		Individual and group mentoring; community posters (Steps 10–11)	x			3
Aug 2026	C2 follow-up assessment				2	
Nov/Dec 2026	C3 endline assessment (1 mo. after intervention ends)					3
June 2027	C3 follow-up assessment					3

### Sample size {14}

Sample size is based on calculations using the outcome measure of proportion of young fathers who apply positive parenting behaviors. Based on the pilot studies on REAL Fathers intervention in Uganda, and to ensure sufficient statistical power of sub-group analyses at the regional level to obtain the largest sample size necessary, we assume only 40% of the young fathers use positive parenting skills at baseline [40]. From pilot studies, the REAL Fathers intervention should improve this proportion by at least 10% (effect size on absolute scale) by the endline [40]. The sample size computation is based on the formula:

$$n=\frac{[2*{\left({Z}_{\alpha /4}+{Z}_{1-\beta }\right)}^{2}*\overline{P }(1-\overline{P })]}{{({P}_{1}-{P}_{0})}^{2}}\times Deff$$

where:

*n* = minimum sample size of individuals required per arm of study

*Z*_α/4_ = the critical value of the standard normal variate at significance level of α/4 (*α* is assumed to be 0.05 or 95% certainty for improvement in the four primary outcome indicators). By setting the critical *Z* value corresponding to an alpha level of 0.0125 (i.e., α/4), our sample size calculation adjusts for multiple outcome testing while maintaining an overall 5% level of statistical significance for the trial.

*Z*_1-β_ = the critical value of the standard normal distribution at a power of 1-β (assumed to be 0.80 or 80% power)

*P*_0_ = the value of positive parenting indicator at the baseline; assumed 40%

*P*_1_ = the value of positive parenting indicator at the endline; assumed to be 50%—a 10-percentage point increase based on pilot studies

$$\overline{P } = \frac{{P}_{1}+{P}_{0}}{2}$$

*Deff* = clustering effect, assumed to be 2.3

Based on the Uganda Bureau of Statistics 2019 population projections and Uganda Demographic and Health Survey 2016 data, only 4% of the male population (16–25 years) are young fathers who are married or cohabiting [6]. According to the latest Uganda population and housing census, the average village size is about 100 households [49]. Thus, no more than 10 young men (16–25 years) will be fathers whose eldest child is below the age of three years. To be on the conservative side, we assume that, on average, only 8 young fathers are eligible for the study from any given village. Let $\overline{m }$ denote this average and suppose *cv* is the coefficient of variation in the number of fathers across the different villages, and ρ is intra-cluster correlation (ICC). Pilot studies on the REAL Fathers intervention approximated ICC of 0.20–0.24 at the village level. Thus:

$$Deff=\left(1+\left(\left({cv}^{2}+1\right)\overline{m }-1\right)\rho \right)=\left(1+\left(\left(0.063+1\right)\times 7-1\right)\right)\times 0.20=2.3$$

Based on the above parameters and an attrition rate of 10% between the baseline and endline, a total of 1872 couple dyads is required per study arm (3744 in total). This translates to 156 couples per district: 78 in each study arm from 6 villages; and 312 couples per geographical region. This makes an assumption of treatment effect size of 10% on indicators at project level and 80% statistical power and 95% certainty (alpha = 0.05).

The study is being conducted across regions characterized by diverse subcultures, both between and within locations. To ensure sufficient power to detect potential heterogeneity in treatment effects across key subcultural groups, we adjusted the sample size accordingly. Drawing on pilot data, we anticipate a moderate level of effect heterogeneity across these subcultures. According to Ford and Norrie (2016) [50] and Kent et al. (2018) [51], moderate heterogeneity corresponds to a coefficient of variation (CV) of approximately 20% in treatment effects. To account for this, we increased the originally planned sample size by 20%, resulting in a target of 2200 participants per study arm.

### Recruitment {15}

A combination of listing and stratified simple random sampling approaches will be applied while selecting study participants. The participants will be recruited within the 24 districts (four districts in each region). In each district, a three-stage sampling design will be applied. In the first stage, two sub-counties will be selected at random considering the urban/rural divide (see Table 7). From the selected sub-counties, up to four parishes will be selected at random. From each selected parish, up to 12 villages will be selected at random and a census/listing of all eligible young fathers as per the inclusion criteria (Table 2, above) will be listed with the help of individuals familiar with the villages (i.e., members of village health teams (VHTs) and village leaders (local council chairpersons or LC1 executives) to generate a sampling frame. A total of 24 villages will be sampled from 4 randomly selected parishes in two randomly selected sub-counties in each district.

**Table 7:** REAL Fathers external evaluation selected sample

	**District**	**Sub-county**	**Randomization**
1	Amuria	Akeriau	Intervention
2	Amuria	Morungatuny	Control
3	Bugiri	Bulidha	Intervention
4	Bugiri	Iwemba	Control
5	Buikwe	Nkokonjeru Tc	Intervention
6	Buikwe	Buikwe Tc	Control
7	Dokolo	Okwalongwen	Intervention
8	Dokolo	Agwata	Control
9	Hoima	Kyabigambire	Intervention
10	Hoima	Kigorobya	Control
11	Ibanda	Keihangara	Intervention
12	Ibanda	Ishongororo	Control
13	Iganga	Nawanyingi	Intervention
14	Iganga	Nakalama	Control
15	Isingiro	Kabuyanda	Intervention
16	Isingiro	Kashumba	Control
17	Kamuli	Bulopa	Intervention
18	Kamuli	Kagumba	Control
19	Katakwi	Katakwi	Intervention
20	Katakwi	Toroma	Control
21	Kayunga	Kangulumira	Intervention
22	Kayunga	Kayunga	Control
23	Kikuube	Bugambe	Intervention
24	Kikuube	Kiziranfumbi	Control
25	Kiryandongo	Bweyale Tc	Intervention
26	Kiryandongo	Kiryandongo Tc	Control
27	Kole	Ayer	Intervention
28	Kole	Bala	Control
29	Lira	Aromo	Intervention
30	Lira	Ogur	Control
31	Luwero	Bamunanika	Intervention
32	Luwero	Katikamu	Control
33	Masindi	Miirya	Intervention
34	Masindi	Pakanyi	Control
35	Mayuge	Imanyiro	Intervention
36	Mayuge	Mpungwe	Control
37	Mbarara	Kagongi	Intervention
38	Mbarara	Kashare	Control
39	Nakasongola	Nabisweera	Intervention
40	Nakasongola	Wabinyonyi	Control
41	Oyam	Ngai	Intervention
42	Oyam	Otwal	Control
43	Rubirizi	Katerera	Intervention
44	Rubirizi	Kirugu	Control
45	Serere	Kateta	Intervention
46	Serere	Kyere	Control
47	Soroti	Tubur	Intervention
48	Soroti	Asuret	Control

A total of 3744 young fathers and their spouses (i.e., couple dyads) will be recruited (1872 per study arm) for this study, or 156 young fathers and their spouses per district. Study participants will be recruited solely through household visits. All young fathers and their spouses willing to follow the study protocol will go through an informed consent process. In a first step, fathers who meet the study’s eligibility criteria will be contacted and briefly informed about the study and invited for an interview. If consent is obtained, the young father’s female partner (either his spouse or cohabitating girlfriend) will be informed about the study and invited for an interview either on the same day or at a later scheduled date. If both the young father and the female partner agree to take part in the study, written informed consent will be obtained from both.

If the couple is not available on the initial day of contact, up to two additional attempts (for a maximum of three visits) will be made to contact the couple on varying days of the week. If the couple declines to participate or cannot be located after the third visit, a new young father will be randomly selected from the village listing. We will use randomization without replacement to reach the required sample.

## Assignment of interventions: allocation

### Sequence generation {16a}

The study will be implemented in 24 districts in six geographical regions of Uganda. As noted above, in each district, a simple random sample of two sub-counties will be taken. These sub-counties will be randomly allocated to either the treatment or control condition (1:1 within each district) using the Mersenne Twister algorithm via the RAND function in Microsoft Excel [52]. For subsequent follow-up studies (midline and endline), all enrolled participants will be re-interviewed at midline and endline surveys. (See Table 7 for sample randomization of sub-counties data).

### Concealment mechanism {16b}

Allocation concealment will be ensured, as the allocation outcome will not be revealed to enumerators at any point and will not be revealed to the implementing partners until the sub-county and fathers and mothers within the sub-county have been recruited into the trial and after all baseline measurements have been completed.

### Implementation {16c}

Study randomization and outcome assessments are done by an evaluation team, while intervention implementation is done by an implementation team of partners, who are blinded to the outcome assessments. After randomization, a list of the sub-counties will be shared with the field implementation team for execution.

## Assignment of interventions: Blinding

### Who will be blinded {17a}

Sub-county stakeholders, trial participants, enumerators, and the implementing partners will be blinded to allocation until after recruitment and baseline measures are complete.

### Procedure for unblinding if needed {17b}

The study design is openly labeled, and so, unblinding of this behavior change intervention will not be possible.

## Data collection and management

### Plans for assessment and collection of outcomes {18a}

Data collection for this phased rollout cluster randomized controlled trial will take place at three points over the course of three cohorts: baseline, endline, and follow-up. Data collection will occur via face-to-face interviews by trained RAs roughly one month before the intervention begins (endline), one month after the intervention ends (baseline), and eight months after endline (follow-up). A mix of male and female RAs (six in each region, 36 in total) will be recruited along the following characteristics and skills: a minimum of a university degree, roughly ages 20–35 years (to foster rapport with the study participants), experience in survey enumeration, and native speakers of the regional languages. All selected RAs will undergo a three-day training on questionnaire administration, consent process and procedure, research ethics, and tracing of respondents. The training will utilize both roleplays, lectures, and PowerPoint presentations. The RAs will also be given an opportunity to try out the data collection tools in the form of roleplays and pre-testing exercises to further build the speed to administer and understand the process of questionnaire administration. Well-performing RAs will be appointed as Field Supervisors (FS) and those who fall below the pass mark of the training test will be discontinued. Six data collection teams will be formed, one for each region. Each data collection team will comprise seven people including one FS, five RAs, and the co-I or other trained research team lead.

Two questionnaires have been developed—one for the fathers and one for the mothers. Both questionnaires have been translated and back-translated from English into six of the local languages spoken by the participants in each region: Ateso, Luganda, Langi, Lusoga, Runyakitara, Runyoro–Rutooro for the respective region. The research team will work with publishers of local newspapers and presenters of radio programs in the respective regions to ensure that the terms used in the surveys reflect the latest context, terms, and vernacular of the local dialects. All translated scripts will be harmonized in a conference of both translators and back translators moderated by the co-I. Additional translations and corrections of the item wording may occur during field training with the RAs before and after piloting with participants to ensure that the meanings of the items in English match the meaning of the local languages, thus helping to ensure more valid responses from the respondents.

Interviews with fathers will last approximately 70 min and interviews with mothers will last approximately 50 min. As much as possible, survey interviews will take place at the respondent’s home, or another private location where the conversation will not be overheard. Survey questionnaires will be programmed for Computer-Assisted Personal Interviews (CAPI) using XML file format in Kobo ToolBox software. Data will be captured through the KoboCollect app (v2024.2.4), an open-source Android application that runs on both mobile devices and computers and allows data export to the Windows operating system [53].All collected data will be stored in a password-protected, web-based database hosted on the KoboToolBox server. Each tablet used for data collection will be password protected to ensure data security. Data capture forms will be designed with inbuilt skips and validation criteria to reduce the chance of inconsistent or incorrect entries and also to enforce that all questions are responded to (with options of ‘don’t know/don’t remember’ and ‘refused’ possible in each question). Data will be checked for completeness and logical consistency before uploading from the mobile devices to the secure Kobo ToolBox server. Any query flagged for errors related to illogical or inconsistent entries will be shared with the responsible RA, and the co-I or field supervisor will consult with and possibly retrain the RA to address the issue, and re-contact the respondent as needed.

### Plans to promote participant retention and complete follow-up {18b}

Mentor involvement with young fathers and female partners is a critical aspect of participant retention; active mentors lead to engaged participants. Thus, to prevent mentor drop-out, the implementing partners publicly acknowledge the mentors’ efforts and involvement at the parish and sub-county level both in-person and in-community media. This leads to an elevated status of the mentors and has been instrumental in maintaining mentors’ sustained engagement and motivation. In addition, the program also keeps mentors and young couples engaged via post-session reflection meetings and in community recognition opportunities on special occasions and in community media. While no direct financial remuneration is provided to either the mentors or young couples, the program ensures that mentors and families are aware of local existing resources and social and economic government initiatives. These include the following: Healthcare services such as access to maternal and child health, reproductive health, HIV/AIDS prevention, and treatment; Education services such as early childhood development, universal primary, secondary and vocational education services; Government initiatives which help families improve their economic stability through financial support and entrepreneurship such as Village Savings and Loan Association (VSLA), Parish Development Model (PDM), Youth Livelihood Program (YLP), Uganda Women Entrepreneurship Program (UWEP), and Graduating to Resilience (GROW); Digital inclusion initiatives, including internet access centers and AI-driven advisory services that promote greater access to information and digital literacy; and linkages registration for national identification cards.

### Data management {19}

Quantitative data from the male and female questionnaires will be collected electronically by trained RAs on tablets programmed using the Kobo ToolBox platform and transferred to the secure Kobo ToolBox cloud account. Back-up data will also be stored on secure servers. Access to the identifiable data is restricted to the Co-I and trial statistician and requires password access. The Co-I and trial statistician are responsible for ensuring the quality of the data, as well as cleaning and anonymizing the survey data before sharing with the PI via encrypted electronic files for further analyses. The PI will not have access to identifiable data. All anonymized data for analysis will be saved in a password-protected server at the University of California San Diego. In addition to these steps, the study will use a number of other data handling and quality control approaches:

- *Standard Operating Procedures Booklet.* The research team will develop a standard operating procedures (SoP) booklet for RAs (enumerators) and Field Supervisors (FS). The SoP booklet will detail survey enumeration instructions and best practices on how to handle challenging scenarios in the field, and provide detailed instructions on how to administer questions that may be difficult for participants to follow, such as those that use a Likert scale.
- *Questionnaire and Enumeration Training and Piloting.* RAs and FS will pilot the item phrasing and data collection platforms during their training to ensure they understand the context and meaning of the items and are familiar with the tablet data collection platform. The SoP booklet will be updated with key insights gained during the RA/FS training and pre-testing of data collection tools.
- *Skip Logic and Validation*: The survey will be programmed with skip logic and validation to ensure valid responses are captured and each question is answered. RAs will also be required to take the GPS reading to validate the respondents’ interview location. This GPS data will be used to verify if RAs are truly at the designated location when collecting data, providing a layer of accountability and reducing the chance of fraudulent reporting. Wireless internet will be activated on all devices and data transmitted in real time to undertake routine and ongoing quality control checks during the data collection period.
- *Field Supervision and Spot Checks*. Each of the six data collection teams will have an FS. Following the overall RA/FS training, the field supervisors will receive additional field supervision training on data quality control and troubleshooting of field-related challenges. The team lead and each FS will also carry out random checks on a range of forms captured by different RAs to ensure completeness of questionnaires. Each FS will also undertake troubleshooting for challenging cases and scenarios in the field. Any issues identified that need retraining will be immediately dealt with. A field supervision meeting with all RAs in the same geographical localities will be undertaken daily, and once a week a general field supervision meeting will be conducted with all sub-teams.
- *Data Quality Control Sample.* We will use a quality control sample of approximately 1% (37 respondents) distributed across the six data collection teams, with approximately 6 respondents per team at each round of data collection. This sub-sample will be re-interviewed on some key questions by a different group of RAs for quality control purposes. If the index of inconsistency is > 20, data collection will be paused until additional data quality control measures can be implemented such as re-training, re-interviewing the respondents, contacting the RAs for explanations, and changing the RAs if necessary.
- *Unique Participant Identifiers*. The database will capture an individual respondent’s unique identifier (ID). The survey is also programmed to generate an automatic ID for all respondents which will reject any duplicate records during data upload. The IDs will include assigned unique variables for district, sub-county, parish, age, and sex, using a coding scheme known only to the co-I, each FS, team leads, and study statistician. Only the co-I and the study statistician will have the final key linking the respondent ID to the name of the individual respondent.

### Confidentiality {27}

All data will be kept confidential and stored on a secure server. Data access will be restricted to those conducting data analysis on the study team, and each will have access to the data only through password protected log-in. Anonymization of data will be carried out by the co-I and study statistician to maintain participant confidentiality. Names of participants will not appear on any of the data collection forms. The statistical modelling required to answer the project’s questions will require only the participants’ identification number and not their names. Upon generation of the data, it will be coded so that the personal identity and individual data from the study participants are traceable only with the code key which will be held by the study researchers; no one else will have access to it. The code key required to transform anonymized data into identifiable data will be stored within the encrypted drives, within encrypted, password protected cloud folders. No identifiable participant information will be reported in publications. Analyses will use pooled data and will be restricted to regional estimates as the smallest unit of analysis. Once analysis is complete, the link and folders will be deleted, and all data collection materials will be handed over for custody or destruction.

### Plans for collection, laboratory evaluation and storage of biological specimens for genetic or molecular analysis in this trial/future use {33}

No genetic or molecular analysis of biological material is planned in this trial.

## Statistical methods

### Statistical methods for primary and secondary outcomes {20a}

The primary analysis will follow an intention-to-treat (ITT) approach, where all study participants will be analyzed according to their original randomization assignment at baseline. To evaluate intervention effects on primary and secondary outcomes, we will employ difference-in-differences (DID) regression models, which are well-suited for analyzing longitudinal data. DID estimation estimates the causal effect of the intervention by comparing changes in outcomes over time between the treatment and control groups while controlling for unobserved time-invariant characteristics, provided the parallel trends assumption and the Stable Unit Treatment Value Assumption (SUTVA) hold. To account for the complex survey design, we will incorporate random effects in the DID regression models to address clustering at the sub-county level (the unit of randomization). Additionally, clustered standard errors will be used to correct for within-individual autocorrelation and intra-cluster correlation (ICC) at the village level. Where necessary, sample survey weights will be applied to adjust for any imbalances in sub-county or district-level characteristics. As noted above in the “Sample size” section on sample size, we apply Bonferroni correction to account for multiple comparisons in primary outcomes while maintaining an overall 5% level of statistical significance for the trial.

Since all trial outcomes are binary, we will use mixed-effects logistic regression models for the primary endline analysis. As a supplementary analysis, we will estimate causal effects in terms of risk differences using mixed-effects linear models, implemented via the *xtdidregress* and *xtreg* commands in Stata. Individual-level observations will be analyzed rather than cluster-averaged data, following methodologies outlined by Hussey & Hughes (2007) and Baio et al. (2015)) [54, 55]. Let ${Y}_{ijk}$ denote an outcome of individual *i* from *j*th subcounty and *k*th region measured at time period *t*, and modelled as:

$$logit(P({Y}_{ijkt}=1)={\beta }_{j}+\lambda {G}_{i}+\alpha {T}_{i}+\gamma {(GT)}_{i}$$

where ${\beta }_{j}$ is the random effects term for the sub-counties (with ${\beta }_{j} \sim N\left(0,{\tau }_{\beta }^{2}\right)$.); $\lambda$ is fixed effect of randomization group (G_*i*_ = 1, if exposed to intervention; and G_*i*_ = 0, otherwise); $\alpha$ is a fixed effect of time (T_i_ = 0 if baseline, T_i_ = 1 if endline), and $\gamma$ is an interaction effect of time and group. Predicted margins will be used to compute the average causal effect or equivalently the ITT estimand.

For follow-up analysis, we will implement a three-time-point panel model to assess the persistence of treatment effects nine months post-intervention closure. Additionally, a two-time-point analysis (comparing endline and follow-up) will be conducted as a sensitivity analysis to evaluate short-termeffects.

### Interim analyses {21b}

We will conduct interim DID regression analyses on the primary and secondary outcomes at the endline and follow-up time periods of Cohort 1 and Cohort 2. Final analyses will be conducted on the full dataset after the end of the intervention for all study cohorts (i.e., after Cohort 3 endline and again at Cohort 3 follow-up).

### Methods for additional analyses (e.g., subgroup analyses) {20b}

For each trial cohort (i.e., data collection cohorts), hierarchical random effects models will be estimated to understand variation or treatment effects heterogeneity at the regional- and sub-county levels. After the full data collection is complete (i.e., after data collection on all three trial cohorts is completed), stratified analyses by region will be conducted to provide policymakers and program implementers more detailed information on intervention effects given hypothesized differentials in socio-cultural norms by geographic region. When assessing outcomes at a regional level, the index indicators will be estimated within 10% precision. In addition, we will conduct exploratory sub-group analyses on key covariates including the following: age of the young father, duration of time living with spouse/partner, age and sex of the child, alcohol consumption, and education level of father and spouse/partner.

### Methods in analysis to handle protocol non-adherence and any statistical methods to handle missing data {20c}

We will conduct per protocol analysis and compare it against intention-to-treat analysis. Dosing data will be captured through participant identifiers that will permit linkages between monitoring and evaluation data in order to permit intervention dose as an exposure variable as opposed to simply including the bivariate exposure of control or intervention. Missing data may arise in different ways including the participants declining to provide the information, dropout of the study, and errors in data capture by the data collectors. The mechanism underlying the missingness will be explored. If missing completely at random, complete case analysis will be done. Missingness at random of outcomes will be handled through augmented inverse probability weighting. Individuals with missing covariates will be included in analysis by including a missing-indicator variable for the covariate as an additional covariate in the regression model.

### Plans to give access to the full protocol, participant level-data and statistical code {31c}

The trial protocol, evaluation indicators, and analysis plan will be registered and published prior to the commencement of data analysis. The data generated and analyzed for this study will be deidentified and, after study completion and publication, can be made available upon reasonable request to the PI (KMB) and subsequent approval by the project management partner, Impact and Innovations Development Centre (IIDC). Access to the data will be granted for research purposes only.

## Oversight and monitoring

### Composition of the coordinating centre and trial steering committee {5d}

The US-based Principal Investigator (PI, KMB) will design and lead the trial, in partnership with the Uganda-based Co-Investigator (co-I, DN), and the project management group (AW, DO) ensuring that the trial runs to time and budget. Data collection must align with the implementation schedule of the three cohorts. This schedule is dependent upon community buy-in and enrollment of eligible fathers. As such, the PI will coordinate closely with the Uganda-based co-I and project management group who are in direct contact with implementing partner organizations and district project officers. The PI, co-I, and project management group meet virtually every week to discuss adherence to the protocol, quality and timing of data collection, and participant safety. In addition, the project management group and implementing partners meet in person and virtually on a weekly basis for supportive supervision, to discuss adherence to the protocol, and to identify any challenges in implementation. The PI and the co-I will be responsible for ensuring trial and data quality.

### Composition of the data monitoring committee, its role and reporting structure {21a}

A formal Data Monitoring Committee (DMC) will not be established for this trial. The study involves a low-risk educational intervention delivered via trained community-based mentors, and there is no anticipated serious harm or adverse effect. Given the low-risk nature of the intervention, the research team has determined that a DMC is not necessary. Instead, the PI and co-Is will work in close collaboration to monitor data collection and trial progression, ensuring adherence to the protocol. Weekly meetings with the research and program team will be established to ensure regular communication on progress. Any deviations from the protocol will be documented and reported.

### Adverse event reporting and harms {22}

The study involves an informal educational intervention for fathers and mothers in Uganda whose eldest biological child is below the age of three years. The intervention is considered low-risk with no anticipated adverse events stemming directly from the intervention. However, it is expected that study enumerators and implementation teams at each study site may identify individuals at risk of or those who are currently experiencing violence and maltreatment. The research and implementing teams are equipped with monitoring tools to document referrals to the nearest health facility or responsible institution and the reasons for referral. These include the following: Child Protection Services through the Child Wellbeing Committees which address cases of child abuse, neglect, and exploitation; Gender-Based Violence (GBV) Prevention and Response through community GBV committees, one-stop centers for survivors, and legal aid services to support GBV survivors; and Social Welfare and Safeguarding services for vulnerable groups such as orphans, elderly persons, and people with disabilities, ensuring they have access to financial and practical assistance.

In the event that a serious adverse event occurs due to the study and intervention, any/all serious adverse events will be reported promptly to the National Council for Science and Technology (UNCST) after ethical review and approval from Makerere University School of Social Sciences Research and Ethics Committee (designated research and ethics committee). Unanticipated problems involving risks to research participants or others will be reported promptly to the UNCST. New information that becomes available which could change the risk/benefit ratio to participants will also be submitted promptly for UNCST notification.

### Frequency and plans for auditing trial conduct {23}

The ethical committees responsible for approval can spontaneously audit trial conduct, independently of investigators and the sponsor.

### Plans for communicating important protocol amendments to relevant parties (e.g., trial participants, ethical committees) {25}

In case of substantial protocol modifications, these will be reported immediately to the following scientific ethics bodies which will review and approve the changes: Makerere University School of Social Science Research and Ethics Committee; Uganda National Council for Science and Technology (UNCST); and University of California San Diego Institutional Review Board. All potential modifications will be tracked and dated.

## Dissemination plans {31a}

Anonymized data will be analyzed, and all results regardless of outcome will be made available by publication in peer-reviewed international journals and presentation at international scientific conferences. In addition, the REAL Fathers Consortium of researchers, implementers, and policymakers will generate on an ongoing basis short briefs and PowerPoint presentations and will ensure findings from baseline, endline, and follow-up are shared with implementing partners and community members; local, regional, and national policymakers; and global public health practitioners.

## Discussion

This phased rollout cluster randomized controlled trial will be the first study of its kind to evaluate the effectiveness of the REAL Fathers intervention, a community-based, gender norms transformative parenting program in six regions of Uganda. We include a large, representative cohort of young fathers and their female partners with children under the age of three across six diverse regions in Uganda. The trial will expand the evidence base on programming that integrates prevention of violence against children and intimate partner violence, alongside the promotion of increased father-child engagement via social support for changes in caregiving norms within families [28]. The trial will measure various outcomes from both fathers and mothers, which were chosen to reflect important aspects of the program theory of change and policy priorities in Uganda. The trial’s primary outcomes are as follows: (1) reduced intimate partner violence; (2) reduced violent discipline of children; (3) increased father-child engagement and play; (4) improved early childhood development. In particular, despite being recognized as a global priority, the impact of violence prevention programs on early child development outcomes has rarely been examined [56]. Given that the earliest years of life are foundational for children’s later life health and wellbeing, understanding the links between family violence, parenting, and early childhood development has significant implications for policy and programming (see, e.g., [36, 57]).

The trial is also powered to stratify analyses by region with the aim of providing policymakers and program implementers more granular information on which to base regional-level programming and policies in response to differentials in socio-cultural norms by geographic region. The trial will also examine additional questions of for whom the intervention works by testing for interaction by age of the young father, duration of time living with a female partner, age and sex of the child, alcohol consumption, and education level of the father and female partner. These outcome data will be complemented by extensive routine monitoring data collected by implementing partners throughout the project cycle to assess fidelity, refine training and delivery, and strengthen implementation quality to maximize effectiveness in achieving the intended outcomes along the theory of change. In addition to generating evidence on ‘what works’ and ‘for whom’ in prevention programming, data generated from this longitudinal cRCT will also allow, for the first time, an examination of household-level violent child discipline and IPV initiated by both male and female partners. This expands the evidence base on heterosexual IPV, which typically focuses on men as perpetrators and women as victims.

This multi-region design and large cohort of couple-dyads enables a rigorous evaluation in a cRCT to measure the effectiveness of the REAL Fathers intervention. The cRCT design and progressive rollout across multiple cohorts will allow for timely adaptations to the program model based on emerging experiences and lessons learned as it scales out to more communities. Findings from this trial will inform future research and programs designed to address intergenerational cycles of violence that impede healthy child development. Our research team will partner with an advisory committee of ministry stakeholders to ensure policy relevance in the national and regional Ugandan policy space to promote sustainability and scale. Ultimately, this study will contribute valuable evidence on how this social and behavior change intervention reduces rates of violence against children, intimate partner violence, and improving child and family wellbeing, while providing an opportunity to investigate the clustering of multiple forms of violence within the household context and their connections to early childhood development.

## Trial status

The protocol is version 1.2 of December 10, 2024. The trial was initiated in November 2023 and recruitment is expected to be complete in December 2025. Enrolment is active at the time of submission.

## Data Availability

After study completion and publication, the trial dataset can be delivered upon reasonable request to the PI (KMB) and subsequent approval by the project management partner, Impact and Innovations Development Centre (IIDC). Access to the data will be granted for research purposes only.

## Appendix

### Model Consent Form

RESPONSIBLE, ENGAGED AND LOVING (REAL) FATHERS OUTCOME EVALUATION STUDY CONSENT FORM (BASELINE-ENDLINE-FOLLOW-UP)—YOUNG FATHER

Impact and Innovations Development Center (IIDC)

University of California, San Diego (UCSD)

Nana Development Consultants Limited (NaNa DCL)

### Introduction

You are invited to take part in this research study. Please take as much time as you need to make your decision. Feel free to talk about your decision with whomever you want, but remember that *the decision to take part in the study, or not take part in the study is yours.*

If you have any questions, you should ask the interviewer who explains this study to you. If you decide to take part in the study, you will be in up to three interviews). Each interview will last about 45 to 60 min. The first interview will take place now and the second and third interviews will take place in about 8 to 12 months from each other. The interviews will take place in a private location wherever you prefer, at a time that is good for you.

### Background and purpose to the study

We are doing this study to find out more about young fathers'parenting skills and ways of disciplining their children as well as the health and well-being of their families. We will use the results to understand how to help make the lives and health of young fathers and their families better.

This study is being sponsored by the Lego Foundation through Impact and Innovations Development Center (IIDC), University of California, San Diego. This means that Lego Foundation through Impact and Innovations Development Center (IIDC) is paying NaNa Development Consultants Limited to conduct this study with *Dr. Symon Wandiembe* and Dennis Nabembezi as the Primary Researchers.

If you decide to do the interview, you will be asked some questions about yourself, such as your age and education. The interviewer will also ask about your thoughts on and experiences with being a father, your thoughts on how men and women should act as parents, and child disciplining. Finally, the interviewer will ask you some questions about your relationship with the mother of your child and about your thoughts and experiences with violence as well as questions about your child’s development. Please note that the types of violent acts that we ask about are in no way endorsed by the researchers, Impact and Innovations Development Center (IIDC), University of California, San Diego or their associates.

Before you decide to participate, we, the interviewers, must tell you about:

1. The study plan – or the reasons why this research is being done, how it will be done, how many people will be involved, and how long it will take.
2. The risks, discomforts – as well as the benefits from the study that we think might happen as well as some that we are aware of.
3. How we keep everything private and confidential.
4. Your rights as a research participant – including knowing that you can end the survey at any time or choose not to answer a particular question if it makes you uncomfortable. And that there will be no repercussions if you decide to stop being in the study.
5. Who else you may contact—other than myself (the enumerator) – if you have questions or concerns about this research.

### Study plan

We will be interviewing about 3,750 young fathers and their spouses or significant others in 24 districts of Uganda across six regions. You are being asked to take part in this study because you live in this community, you are a father with a child under the age of three years old, living with your wife or girlfriend, and you are between the ages of 16 and 25.

If you decide to take part in this study, you will be randomly placed into one of two groups—this means that your placement in one of these two groups will be by chance. One group will begin taking part in a Young Fathers Mentoring Initiative very soon, and the other group will begin taking part in this Young Fathers Mentoring Initiative in 8 to 12 months from now – after the first group has finished. This Initiative is a program that includes mentoring sessions and group discussions with young fathers. In these activities, the group will talk about topics like parenting, communicating with your wife, and solving arguments without violence. We can only tell you which group you will be in after you agree to be in the study. If you decide to be in the study, no matter which group you are assigned to, you will take part in three interviews to learn more about your experiences as a young father. Each interview will last about 45 to 60 min. The first interview will take place now and the second and third interviews will take place in about 8 to 12 months after each subsequent interview.

### Risks

There are a few risks that are possible if you agree to be in this study. It is possible that talking about some of the personal topics in the interview will make you think about things or memories that could make you feel uncomfortable or sad. Know that if any topic or question makes you feel uncomfortable or sad, you will not need to respond to those questions. You will not have to talk about topics that embarrass you. And at any time, you can ask to change the topic, stop the interview, or ask to stop being in the study. After the interview, if you want advice or information about any of the topics we discuss, let the interviewer know. He or she can help you find a trained person who can provide you with information, advice, or services.

It might also be embarrassing to you if other people in the community hear about the things you say during the interview. We don’t want that to happen, so we will not share anything you tell us during the interview with anyone else in your community. We will also make sure the interview is conducted in a private place where no one will overhear your responses. There may also be other risks that the study team is not aware of. We will inform participants if any new risks other than the ones we have noted here become apparent.

### Benefits

If you agree to take part in this study, there will be no direct benefit to you. That means you will not receive any money or gifts for being in the study. But we believe that the information collected in this study will help us and other groups like us create programs that will make the lives of families in your community and other communities in Uganda better. In case of any cost for participating in the study such as transport these costs will be reimbursed.

### Confidentiality

Any information that you share with us will be kept private. Your name will not appear on any notes or on the interview guide. We will do everything possible to keep your information private but it is impossible to guarantee absolutely that no one will see the information. In order to keep information about you as safe as possible, as soon as we finish each interview, we will take the notes and interview guide to a locked and safe place. Only interviewers on the study will be able to see them. When we leave the village, we will take the notes and interview guide and put them in a locked and safe place again. The answers that you give us and all notes will be put on a special computer that no one can use except the interviewers who are doing the study.

Your name or any information that could identify you or your family will not be included in any reports or publications from this study. But note that the University of California, San Diego Institutional Review Board can access your study records if there is a need to review the data for any reason.

### Your rights as a research participant

Participation in this study is voluntary at all times. You can choose not to participate at all or to leave the study at any point. If you decide not to participate or to leave the study, there will be no bad effect on your relationship with the interviewer, your relationship with Lego Foundation, Impact and Innovations Development Center (IIDC) and University of California, San Diego or their associates or any other negative results for you. If you receive any services from Lego Foundation, Impact and Innovations Development Center (IIDC) and University of California, San Diego, they will not be affected in any way. If you choose to leave the study and not participate in interviews, you can still be in the Young Fathers Mentoring Initiative.

During the interview, you do not have to answer all the questions. Only respond to questions that you are comfortable talking about, and tell the interviewer if you don’t want to answer a question. If you decide that you no longer want to be in the study, just tell the interviewer. In that case, the information we already have through your participation will be included in the data analysis and final report for this study*,* unless you tell us that don’t want it to be included.

### Questions and concerns

If you have questions or concerns about the study, you may contact Dr. Symon Wandiembe or Dennis Nabembezi (Lead Local Principle Investigator) at NaNa Development Consultants Limited, located at Plot 164, NaNa Plaza, Kayunga Trading Center, Hoima Road, telephone: 0773263216/0772568359 or professors at University of California San Diego: Dr. Rebecka Lundgren, PhD (rlundgren@ucsd.edu) and Dr. Kathryn M. Barker, PhD (k1barker@ucsd.edu). You can also contact Moses Komagum, Head of Programmes, Impact and Innovations Development Center (IIDC), at Plot 175/6 Kyadondo II Road, Kagugube Zone, Makerere, Kampala, telephone 0782227262. Please call Dr. Stella Neema, the chairperson of Makerere University College of Humanities, School of Sciences Ethics and Research Committee (REC) on 0784 285,402 if you have any questions about your rights as a research participant or Uganda National Council of Science and Technology on 0414–705500. “Now that I have read this information about the study and the research, do you have any questions for me?”

Response option (SELECT ONE):

Yes

No

Are you interested in participating in this study? (IF YES IS SELECTED, YOU WILL NEXT GET THE RESPONDENT’S SIGNATURE ON THE INFORMED CONSENT FORM. IF NO IS SELECTED, THIS WILL END THE INTERVIEW.)

Response options (SELECT ONE):

Yes – signature

No -> END

### Respondent consent and signature (obtain witness signature only if participant cannot write)

Signing this document means that the research study, including the above information, has been described to you orally and you understand the contents of the consent form, and that you voluntarily agree to participate in the study.

I understand all the information in the consent form. I have been provided an opportunity to ask questions and I have been provided all answers to questions asked. I freely and voluntarily agree to take part in the study.

[required] Name of participant/witness:

[required] Today’s Date:

[required] Signature:

### Enumerator confirmation and signature

I have fully explained to the participant the study purpose and procedures, the possible risks and benefits, and that participation is completely voluntary. I have invited the participant to ask questions and I have given complete answers to the best of my ability to all of the participant’s questions.

[required] Name of the Person obtaining consent:

[required] Today’s Date:

[required] Signature:

## Abbreviations

Co-I: Co-Investigator
cRCT: Cluster randomized controlled trial
DID: Differences-in-differences
DMC: Data Monitoring Committee
ECD: Early childhood development
FS: Field Supervisor
GEH: Center on Gender Equity and Health at the University of California San Diego
ICC: Intra-cluster correlation
IP: Implementing partner
IPV: Intimate partner violence
ITT: Intention-to-treat
LCI: Local Council 1
M&E: Monitoring and evaluation
MIS: Monitoring Information System
PI: Principal Investigator
RA: Research Assistant
REAL Fathers: Responsible, Engaged and Loving Fathers
SoP booklet: Standard Operating Procedures booklet
UNCST: Uganda National Council for Science and Technology
VAC: Violence against children
VHT: Village Health Team
VSLA: Village Savings and Loan Association
